# The Human α-Lactalbumin Molten Globule: Comparison of Structural Preferences at pH 2 and pH 7

**DOI:** 10.1016/j.jmb.2009.09.025

**Published:** 2009-11-27

**Authors:** Heike I. Rösner, Christina Redfield

**Affiliations:** Department of Biochemistry, University of Oxford, South Parks Road, Oxford OX1 3QU, UK

**Keywords:** α-LA, α-lactalbumin, 4SS α-LA, α-LA containing the four native disulfide bonds, all-Ala α-LA, α-LA containing no disulfide bonds, [28–111] α-LA, α-LA containing a single disulfide bond between Cys28 and Cys111, L11P, [28–111] α-LA with a proline replacing a leucine at position 11, Q117P, [28–111] α-LA with a proline replacing a glutamine at position 117, HSQC, heteronuclear single quantum coherence, human α-lactalbumin, molten globule, protein folding, pH dependence, NMR

## Abstract

Structural investigations of molten globules provide an important contribution towards understanding protein folding pathways. A close similarity between equilibrium molten globule states and kinetic species observed during refolding has been reported for several proteins. However, the experimental conditions, and in particular the pH, under which the equilibrium and kinetic species are studied often differ significantly. For human α-lactalbumin (α-LA), the equilibrium molten globule is most often studied at pH 2, the so-called A-state, while kinetic refolding experiments are performed at neutral pH. α-LA contains a large number of acidic amino acid residues that may influence the properties of the molten globule differently at low and neutral pH. In this study, we investigate the structural preferences of the α-LA molten globule at pH 7 at the level of individual residues using nuclear magnetic resonance spectroscopy and compare these data with previous results obtained at pH 2. We show that differences exist in the conformational ensemble that describes the α-LA molten globule at these two pH values. The molten globule at pH 7 is generally less stable than that at the low pH A-state. Most notable are differences in the stability of structure for the C-helix and the calcium-binding loop that precedes it and differences in the contribution of long-range hydrophobic contacts between the N-terminal and C-terminal regions of the α-domain to the stability of the molten globule. Our results are discussed in the context of previous studies of the α-LA molten globule and can be used to reconcile apparent discrepancies in published data relating to the C-helix. In the light of our results, the low pH A-state may not be the best model for the kinetic molten globule observed during refolding of α-LA. The pH-dependent effects reported here for α-LA may be of relevance in comparisons of equilibrium and kinetic molten globules of other proteins.

## Introduction

Molten globules can be regarded as a thermodynamically distinct state of proteins,^1^ showing properties of both native and denatured proteins.^2,3^ Generally, molten globules are observed as intermediates on the protein folding pathway and, hence, are often only transiently populated during protein folding. In some cases, however, molten globules exist as stable conformers under mildly denaturing conditions, such as acidic pH.^3^ The role of molten globules has also been expanded to include physiological processes including membrane penetration^4^ and ligand release.^5^ Convincing evidence exists for a close resemblance between equilibrium molten globules and the transient kinetic folding intermediates for proteins including apomyoglobin and α-lactalbumin (α-LA).^6–8^ However, the experimental conditions, and in particular the pH, under which the equilibrium and kinetic species are studied often differ significantly. For α-LA, the equilibrium molten globule is most often studied at pH 2, the so-called A-state, while kinetic refolding experiments are performed at neutral pH. α-LA contains a large number of acidic amino acid residues that may influence the properties of the molten globule at low and neutral pH; at low pH, these residues will be uncharged, while at neutral pH, above their p*K*_a_, they will carry a negative charge. In this study, we investigate the structural preferences of the α-LA molten globule at pH 2 and pH 7 at the level of individual residues using nuclear magnetic resonance (NMR) spectroscopy.

**Fig. 1:**
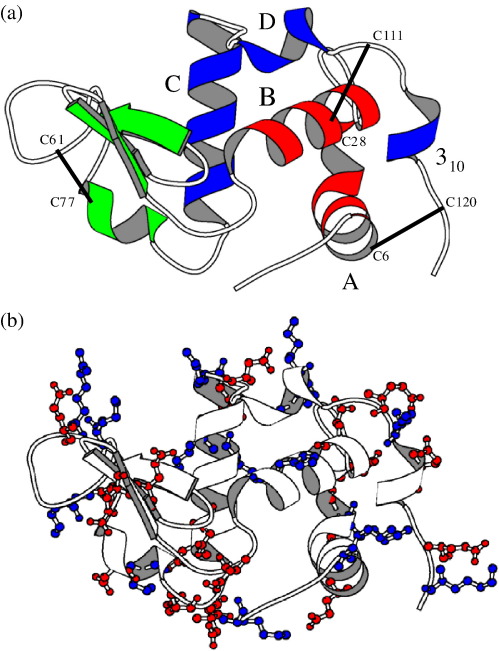
Schematic representation of the native structure of human α-LA. (a) The α-domain is shown in red (residues 1–39) and blue (residues 82–123), and the β-domain (residues 40–81) is shown in green. The disulfide bonds present in 4SS and [28–111] α-LA are indicated and the α-domain helices are labeled. (b) Acidic (red) and basic (blue) residues are shown in a ball-and-stick representation. The diagrams were generated using MOLSCRIPT^9^ and the X-ray coordinates for the native protein.^10^

Human α-LA is a 14-kDa protein whose native structure is divided into two domains: the α-domain is largely helical and the β-domain has a significant β-sheet content (Fig. 1). It is generally agreed that the human α-LA molten globule has a bipartite structure; the α-domain has native-like helical structure while the β-domain is largely unfolded.^11–15^ A proline mutagenesis study, carried out at pH 8.5, suggests that the A, B, D, and C-terminal 3_10_ helices of the α-domain can be disrupted individually without the loss of other helical structure.^16^ Introduction of a proline into the C-helix does not lead to the expected loss of helical structure, as monitored by circular dichroism (CD), and it was concluded that the C-helix is not formed in the α-LA molten globule. In contrast to this, NMR and mass spectrometry studies of backbone amide protection in the α-LA molten globule at pH 2 showed significant protection of amides in the C-helix, indicating clearly that this helix is formed.^17,18^ These contradictory results suggest the possibility of differences in the α-LA molten globules at high and low pH.

Only one study to date has focused on comparing the α-LA molten globule at pH 2 and pH 7.^19^ In particular, the roles of the C6-C120 disulfide bond and the β-domain were investigated using full-length α-LA with the four native disulfide bonds, a three-disulfide variant lacking the C6-C120 disulfide bond and a peptide composed of residues 1–38, containing the A- and B-helices, cross-linked via the C28-C111 disulfide bond to residues 95–120, containing the D-helix and the C-terminal 3_10_ helix (ABD_95–120_). Overall structural features of these proteins were studied using ‘low-resolution’ spectroscopic methods including near- and far-UV CD and fluorescence. In addition, the equilibrium unfolding in urea was monitored to give a measure of overall stability. The study reveals that the correct pairing of the C6-C120 disulfide bond is more important for the formation of secondary structure at pH 2 than at pH 7. In contrast, the β-domain was found to play a significant role for the overall stability at pH 7 but not at pH 2.^19^

To date, NMR studies of the α-LA molten globule have largely focused on the classical A-state, which is formed at pH 2, as it is the most stable and readily accessible form of the α-LA molten globule. The NMR spectrum of the α-LA molten globule is characterized by broad resonances for most of the backbone amide groups, making direct study of the molten globule difficult.^15,20–22^ This line broadening is interpreted as arising from constrained conformational fluctuations on the microsecond to millisecond time scale.^15,20,23^ Resonances broadened to such an extent that they are not observable in ^15^N–^1^H heteronuclear single quantum coherence (HSQC) spectra of α-LA correspond to residues that are in compact folded regions of structure in the molten globule, whereas sharp and well-resolved peaks observed in the HSQC spectra correspond to highly dynamic and largely unfolded regions of the protein.^15,21,22^ In titrations with urea, the number of well-resolved resonances observed in the HSQC spectrum is found to increase as the concentration of urea is raised. This stepwise appearance of peaks is consistent with noncooperative urea-induced unfolding of the molten globule. Peaks that appear at the lowest concentrations of urea correspond to residues of the α-LA molten globule that are in less stable regions of structure. The residues most resistant to urea are, in the native structure of α-LA, clustered together in the core of the protein, indicating that this is the most stable region of the molten globule. This approach has been used previously to study in detail the urea-induced unfolding at pH 2 of structure in the molten globules of human α-LA containing all four native disulfide bonds (4SS α-LA) and variants of α-LA containing only the C28-C111 disulfide ([28–111] α-LA) or lacking all disulfides (all-Ala α-LA).^15,21,22^ Here, we have applied this NMR approach to characterize these molten globules at the level of individual residues at pH 7. Comparison of the results at pH 2 and pH 7 allows the differences in the two molten globule species to be defined for the first time at the residue-specific level.

## Results

### Overall helical secondary structure in the α-LA molten globule

**Fig. 2:**
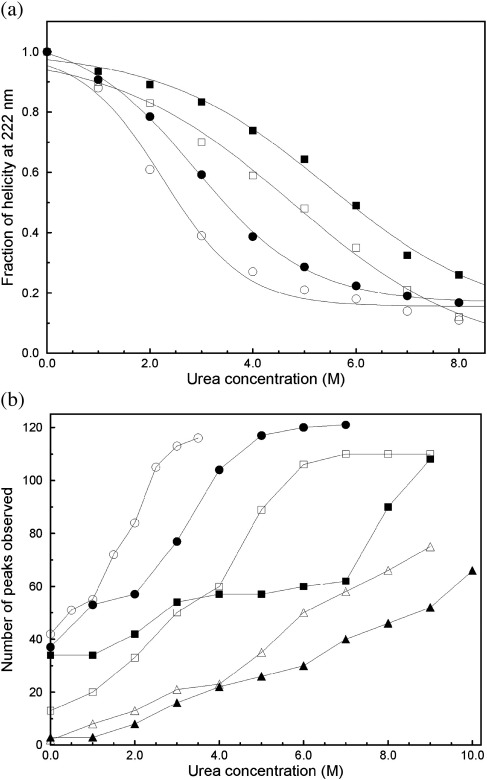
Urea-induced unfolding of the α-LA molten globule at pH 2 and pH 7. (a) Loss of helical secondary structure was monitored by far-UV CD spectroscopy at 222 nm for all-Ala α-LA at pH 2 (●), all-Ala α-LA at pH 7 (○), [28–111] α-LA at pH 2 (▪), and [28–111] α-LA at pH 7 (□). All transitions were normalized so that a value of 1.0 corresponds to the helical content of the molten globule in the absence of urea. (b) The number of peaks visible in the HSQC spectrum is shown as a function of urea concentration for all-Ala α-LA at pH 2 (●), all-Ala α-LA at pH 7 (○), [28–111] α-LA at pH 2 (▪), [28–111] α-LA at pH 7 (□), 4SS α-LA at pH 2 (▴), and 4SS α-LA at pH 7 (▵).

Horng *et al.* have previously studied the pH dependence of the stability of helical structure for the molten globule state of human α-LA containing the four native disulfide bonds (4SS α-LA), for a three-disulfide species lacking the C6-C120 bond, and for their peptide model of the molten globule minimum core (ABD_95–120_).^19^ They found that for all three proteins, helical secondary structure was more resistant to urea-induced unfolding at pH 2 than at pH 7. For example, the unfolding midpoint for 4SS α-LA was found to decrease from 6.9 to 4.6 M urea when the pH was increased from 2 to 7. The ABD_95–120_ peptide was less stable than the intact protein with unfolding midpoints of 4.9 and 2.7 M urea at pH 2 and pH 7, respectively. We have extended these studies to the single-disulfide species, [28–111] α-LA, and to all-Ala α-LA, which lacks all four disulfides. The loss of helical structure was monitored using far-UV CD spectra over a range of urea concentrations (Fig. 2a). The unfolding midpoint urea concentrations, *C*_m_ values, are summarized in Table 1. These two α-LA variants show a similar trend to that observed by Horng *et al.* with decreased stability at pH 7, but the difference in stability observed is smaller than that reported for 4SS α-LA.^19^

### NMR spectra of the α-LA molten globules in urea at pH 7

We have demonstrated previously that NMR spectroscopy can be used to monitor the stepwise, noncooperative unfolding of the α-LA molten globule and that this technique can provide important insights at a residue-specific level for the molten globule.^15,21,22^ Previous studies of all-Ala, [28–111], and 4SS α-LA have been carried out at pH 2. These experiments have been extended here to characterize these species in detail at pH 7.

All-Ala and [28–111] α-LA are not able to bind calcium and exist as molten globules at pH 7.^24^ 4SS α-LA with the native N-terminal amino acid sequence (K1-Q2-F3) adopts a folded, native conformation at pH 7.0 in both the presence and absence of Ca^2+^.^25^ The native state of recombinant human α-LA, with an N-terminal methionine preceding K1, is significantly less stable at pH 7 in the absence of Ca^2+^ and adopts a molten globule conformation at 20 °C in the absence of salt.^26^ Thus, by using ^15^N-labeled recombinant 4SS α-LA with an N-terminal methionine, we are able to study the molten globule of 4SS α-LA at pH 7.

**Fig. 3:**
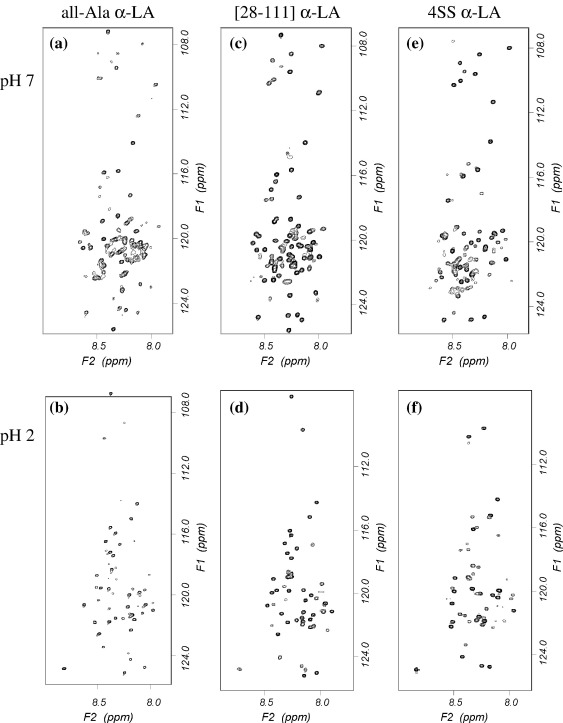
Gradient-enhanced ^1^H–^15^N HSQC spectra of the uniformly ^15^N-labeled human α-LA variants in 95% H_2_O/5% ^2^H_2_O. (a) all-Ala α-LA in 2 M urea at pH 7, (b) all-Ala α-LA in 2 M urea at pH 2, (c) [28–111] α-LA in 5 M urea at pH 7, (d) [28–111] α-LA in 5 M urea at pH 2, (e) 4SS α-LA in 9 M urea at pH 7, and (f) 4SS α-LA in 9 M urea at pH 2.

HSQC spectra collected for all-Ala, [28–111], and 4SS α-LA at pH 7 in increasing concentrations of urea show the same general trends observed by far-UV CD (Fig. 3). The C28-C111 disulfide bond contributes substantially to the stability of the molten globule. This trend can be seen clearly in Fig. 2b where the total number of peaks observed at each urea concentration is plotted as a function of the urea concentration. A similar number of peaks are observed for [28–111] α-LA in 5 M urea as are observed for all-Ala α-LA in 2 M urea. HSQC spectra for all-Ala α-LA collected in 2 M urea at pH 7 and pH 2 are compared in Fig. 3a and b. Peaks corresponding to 84 and 57 residues are observed at pH 7 and pH 2, respectively, indicating that the all-Ala α-LA molten globule is significantly more unfolded at the higher pH. HSQC spectra for [28–111] α-LA collected in 5 M urea at pH 7 and pH 2 are compared in Fig. 3c and d. Peaks corresponding to 89 and 57 residues are observed at pH 7 and pH 2, respectively, again indicating that the [28–111] α-LA molten globule is less stable at the higher pH.

Complete unfolding of the 4SS α-LA molten globule at pH 7 is more difficult to observe by NMR. At pH 2, significantly elevated temperature (up to 50 °C) and the use of 8 M guanidine HCl were required for the observation of a complete set of unfolded peaks.^15^ High temperatures could not be used at pH 7 due to the significantly higher intrinsic amide exchange rates; an increase in temperature resulted in a loss of peaks from the spectrum rather than an increase as observed at pH 2. In 5 M urea, peaks for 35 and 26 residues were observed for 4SS α-LA at pH 7 and pH 2, respectively. This is a significantly smaller number of peaks than reported above for [28–111] α-LA in 5 M urea; this indicates that 4SS α-LA is significantly more stable than [28–111] α-LA. This result is in contrast to the CD data that show more similar stabilities for the two proteins. This apparent inconsistency can be explained by the observation that many of the additional peaks observed for [28–111] α-LA arise from residues located in the β-domain of the protein, which does not contribute to ellipticity at 222 nm. In 9 M urea, peaks for only 75 and 52 of the 121 expected residues are observed at pH 7 and pH 2, respectively (Fig. 3e and f). The lack of complete unfolding for 4SS α-LA limits, to some extent, the analysis that can be carried out for this species at pH 7.

### Stepwise unfolding of the molten globules at pH 7

**Fig. 4:**
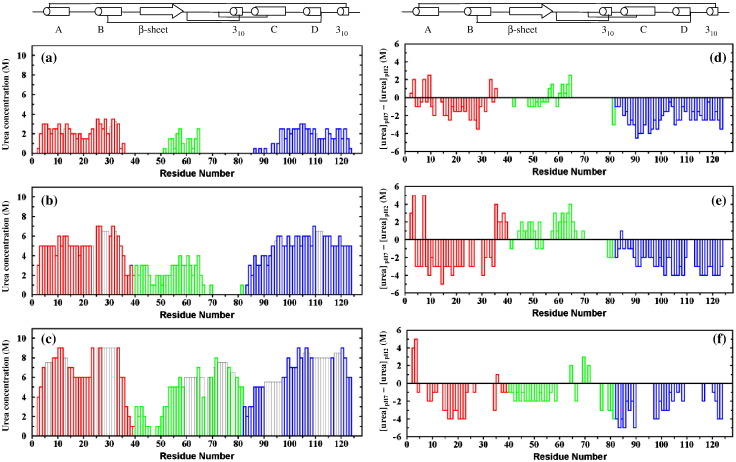
Histograms summarizing the urea-induced unfolding transitions monitored by NMR at pH 7. (a–c) The height of the bars represents the urea concentration at which a peak is first observed for each residue in the HSQC spectra. All spectra were recorded at 500 MHz in 95% H_2_O/5% ^2^H_2_O, at 20 °C. Gray bars indicate residues for which analysis was hampered by peak overlap or the lack of a peak assignment; for these residues, the height of the bar is the average value found for surrounding residues. (a) all-Ala α-LA, (b) [28–111] α-LA, (c) recombinant 4SS α-LA. (d–f) Difference in the urea concentration at which a peak first appears for each residue at pH 7 and pH 2 ([urea]_pH 7_ − [urea]_pH 2_). (d) all-Ala α-LA, (e) [28–111] α-LA, (f) recombinant 4SS α-LA. Differences are only shown for residues that give resolved peaks at both pH 2 and pH 7. The secondary structure found in native α-LA is summarized above. The α-domain is shown in red (residues 1–39) and blue (residues 82–123), and the β-domain (residues 40–81) is shown in green.

The trends described above for all-Ala, [28–111], and 4SS α-LA can be analyzed at the level of individual residues because peaks in the HSQC spectra can be assigned. The observed pattern of unfolding for each protein is plotted as a function of amino acid sequence in Fig. 4a–c. The difference in the urea unfolding pattern is also plotted in Fig. 4d–f to highlight the differences observed between pH 7 and pH 2; a negative bar represents destabilization at pH 7.

The unfolding behavior of 4SS, [28–111], and all-Ala α-LA at pH 7 shows the same general trends as observed previously at pH 2. α-LA shows a bipartite pattern of unfolding; residues in the α-domain are, in general, more resistant to unfolding by urea than β-domain residues. The presence of disulfide bonds in 4SS α-LA leads to stabilization of β-domain residues close in sequence to the cysteines. At pH 7, β-domain residues in all-Ala and [28–111] α-LA are stabilized relative to α-domain residues; this is highlighted in the difference histograms shown in Fig. 4d and e. In these proteins, residues in the region of 55–65 in the β-domain have similar stability to some residues in the α-domain (Fig. 4a and b). In the absence of the β-domain disulfides, many residues in the β-domain, in particular residues 65–81, are completely unfolded at both pH values; hence, the stabilities of this region cannot be compared at the two pH values. The first half of the β-domain shows different behavior in the three α-LA variants; an average stabilization by ∼ 1 M urea is observed for residues 40–64 in [28–111] α-LA at pH 7 while an average destabilization by ∼ 1.5 M urea is observed for residues 40–58 in 4SS α-LA.

In general, residues in the α-domain are less stable at pH 7 than at pH 2. In all-Ala α-LA, α-domain residues from the A-, B-, C-, and D-helices and from the C-terminal 3_10_ helix are destabilized by an average of ∼ 0.5, 2, 3, 2, and 2 M urea, respectively. In [28–111] α-LA, larger destabilization at pH 7 is observed with average values of ∼ 2.5, 3, 2, 3.5, and 3.5 M urea for the A-, B-, C-, and D-helices and the C-terminal 3_10_ helix, respectively. Destabilization of the helices in 4SS α-LA is more difficult to analyze because of the significant number of residues in all five helices that are not observed in 9 M urea at pH 7. The N-terminus of the α-domain is an exception to the general trend of destabilization at pH 7; Q2 and F3, which were completely unfolded in the absence of urea at pH 2, are missing from the spectrum at pH 7. Peaks for Q2 and F3 appear in 0.5 and 2 M urea in all-Ala α-LA, in 3 and 5 M urea in [28–111] α-LA, and in 4 and 5 M urea in 4SS α-LA. At pH 7, a peak for K1 was not observed; this is likely to be due to a high intrinsic amide exchange rate.

The C-helix is the least stable helix in all three α-LA variants at pH 2.^15,21,22^ The loss of stability of this helix at pH 7 means that in all-Ala α-LA, four C-helix residues give rise to observable peaks in the absence of urea and the helix is completely unfolded in 2.5 M urea. In [28–111] α-LA, the C-helix is more stable, but nevertheless seven residues are visible in 4 M urea at pH 7 in contrast to only three residues at pH 2. Significant destabilization at pH 7 is also observed in all three α-LA variants for residues 79–85 that precede the C-helix. In the native protein, several of these residues (K79, D82, and D84) are involved in calcium binding along with D87 and D88 from the C-helix.^10^ The effect is particularly large in 4SS α-LA with an average destabilization of 4 M urea for residues 79–85. A less pronounced destabilization of ∼ 1.5 M urea is observed for all-Ala and [28–111] α-LA.

### The proline variants L11P and Q117P at pH 7

Two variants of [28–111] α-LA, containing proline substitutions in the A-helix and the C-terminal 3_10_ helix (L11P and Q117P), have been studied previously at pH 2.^22^ The L11P variant showed unfolding of the A-helix and AB loop in the absence of urea. The remaining B-, C-, and D-helices and the C-terminal 3_10_ helix were able to form a stable, compact core in the absence of the A-helix; this core was destabilized by ∼ 3 M urea compared to [28–111] α-LA. The Q117P variant also showed a general destabilization of the B-, C-, and D-helices by ∼ 2 M urea, and the C-terminal 3_10_ helix, containing the Q117P mutation, was destabilized by 3–5 M urea relative to [28–111] α-LA. Interestingly, the A-helix and the AB loop at the N-terminus of the α-domain were also destabilized by 3–5 M urea in Q117P. It was concluded that long-range contacts between the N- and C-terminal regions were crucial for the stability of the A-helix in the [28–111] α-LA molten globule at pH 2.^22^

**Fig. 5:**
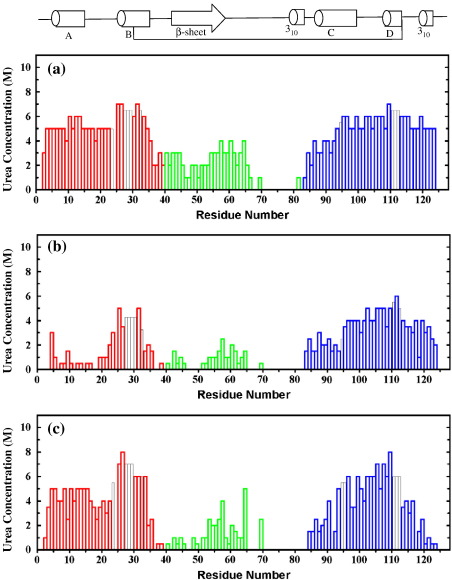
Histograms summarizing the urea-induced unfolding transitions monitored by NMR at pH 7. The height of the bars represents the urea concentration at which a peak is first observed for each residue in the HSQC spectra. All spectra were recorded at 500 MHz in 95% H_2_O/5% ^2^H_2_O, at 20 °C. Gray bars indicate residues for which analysis was hampered by peak overlap or the lack of a peak assignment; for these residues, the height of the bar is the average value found for surrounding residues. (a) [28–111] α-LA, (b) L11P α-LA, (c) Q117P α-LA. The secondary structure found in native α-LA is summarized above. The α-domain is shown in red (residues 1–39) and blue (residues 82–123), and the β-domain (residues 40–81) is shown in green.

Here, we have investigated the urea unfolding behavior of L11P and Q117P at pH 7; the results are summarized in Fig. 5. The L11P mutation at pH 7 leads to significant unfolding of the A-helix and the AB loop as observed previously at pH 2.^22^ The B-, C-, and D-helices and the C-terminal 3_10_ helix are able to form a stable core, in the absence of the A-helix, but this is destabilized by ∼ 2 M urea compared to the helical core in [28–111] α-LA pH 7. The Q117P mutation leads to significant destabilization of the C-terminus compared to pH 2; peaks from residues 118 to 123 are observed at urea concentrations of 0.5 to 2.5 M urea in contrast to concentrations of 4 to 6 M urea at pH 2. Interestingly, the A-, B-, C-, and D-helices in Q117P at pH 7 show a very similar stability to these regions in [28–111] α-LA at pH 7. Therefore, in Q117P, unfolding of the A-helix and AB loop does not appear to be coupled to unfolding of the C-terminus, in contrast to previous observations at pH 2.^22^

## Discussion

We have used NMR spectroscopy to study the urea-induced unfolding of the molten globule states of 4SS, [28–111], and all-Ala α-LA at pH 7. The residue-specific information obtained has been compared to results obtained previously at pH 2.^15,21,22^ Overall, we observe that the α-domain of these proteins is destabilized as the pH is increased from 2 to 7. In [28–111] α-LA, many β-domain residues are stabilized at the higher pH, whereas in 4SS α-LA, most of these residues are destabilized at pH 7. The pH-dependent changes in stability observed for the molten globule must arise from differences in the number and distribution of charged amino acids at the two pH values. On the other hand, the differences in pH-dependent changes in stability observed between the three variants must arise from the way that disulfide bonds influence the relative positions of charged groups.

**Fig. 6:**
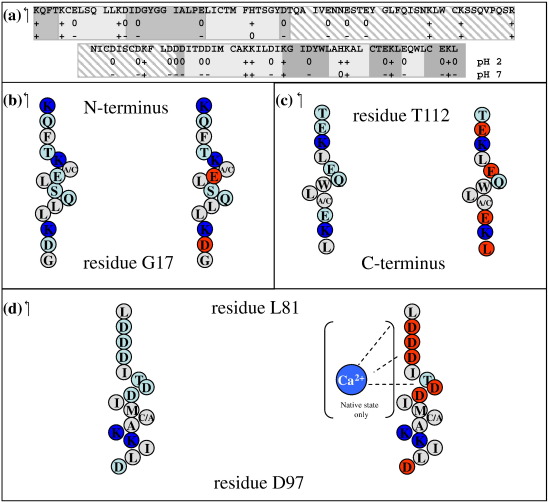
Schematic representations of the distribution of charged residues in the human α-LA sequence. (a) The α-domain is shown in solid gray; darker gray boxes highlight the location of helices in the native state. The β-domain is shown as hashed gray boxes. The charge expected at pH 2 and pH 7 for the acidic and basic residues is shown as + (positive charge), − (negative charge), and 0 (neutral). (b–d) Cartoons of the individual α-domain helices. In each panel, schematic representations of the helix are shown for pH 2 (left) and pH 7 (right). Positively charged residues are highlighted in dark blue, negatively charged residues are colored red, hydrophilic residues are colored light blue, and hydrophobic residues are in gray. (b) A-helix, (c) C-terminal 3_10_ helix, (d) C-helix. The Ca^2+^ ion, only present in the native state, is highlighted in blue.

The distribution of ionizable residue in α-LA is summarized in Figs. 1b and 6a. α-LA contains 12 lysine, 1 arginine, and 2 histidine groups in addition to the N-terminal NH_3_^+^ group; the α-domain contains 12 of these residues, while the β-domain has only 4 basic residues. α-LA contains 12 aspartic acid and 8 glutamic acid residues in addition to the C-terminal carboxyl group; 16 of these acidic groups are located in the α-domain, while only 5 are found in the β-domain. At pH 2, these acidic amino acids will be protonated and α-LA will have an overall charge of + 16; the α-domain has a charge of + 12, while the β-domain has a charge of + 4. At pH 7, the 21 deprotonated acidic residues and the 14 basic residues (histidine is assumed to be neutral at pH 7) give α-LA an overall charge of − 7; the α-domain has an overall charge of − 6, and the β-domain has a charge of − 1. Thus, at low pH, α-LA has an overall positive charge, while at pH 7, it is negatively charged.

In contrast to the general destabilization observed for the α-domain, the N-terminal residues are stabilized at pH 7. In the native state, a salt bridge exists between the negatively charged carboxyl group of E7 and the positively charged side chain of K1. A similar interaction in the molten globule may restrict the backbone conformation and stabilize some residual structure at the N-terminus at pH 7, resulting in broadening of peaks for Q2 and F3 in the absence of urea. At pH 2, where E7 is uncharged, the salt bridge is not formed and the N-terminal residues, K1, Q2, and F3, are completely unfolded.

The β-domain shows different behavior in the three variants studied. In [28–111] α-LA and, to some extent, in all-Ala α-LA, residues in the first half of the β-domain are stabilized at pH 7. In contrast, destabilization is observed in 4SS α-LA. This difference in behavior is likely to arise because of the presence of the C61-C77 disulfide bond within the β-domain and the C73-C91 disulfide linking the two domains. Positive and negative charges are not distributed evenly in the β-domain (Fig. 6a). The first half of the domain contains three glutamic acid residues, E43, E46, and E49, while the second half of the domain contains a number of positively charged residues, K58, K62, R70, and K79, and two acidic residues, D74 and D78. All-Ala and [28–111] α-LA do not have any disulfide bonds restricting the conformation within the β-domain. Thus, at pH 2 and pH 7, β-domain residues, which are relatively unstructured in these variants, are able to sample a conformational ensemble that optimizes electrostatic interactions, and this ensemble is likely to differ at the two pH values. The overall charge of the β-domain is lower at pH 7 than at pH 2 (− 1 at pH 7 and + 4 at pH 2). The balancing of charges at pH 7 coupled to the freedom of the backbone to sample conformational space may lead to the formation of stable interactions in the absence of urea. At pH 2, there may be greater repulsion of the positively charged residues leading to a lower stability compared to pH 7. In 4SS α-LA, the C61-C77 and C73-C91 disulfide bonds will restrict, to some extent, the conformations that may be sampled by the β-domain. At pH 7, this may restrict the formation of stabilizing contacts between the three closely spaced negatively charged glutamate residues in the first half of the β-domain and positively charged residues in the second half of the domain or in the α-domain. This may lead to a general destabilization of this domain at pH 7 compared to pH 2.

The C-helix and residues from the calcium-binding loop that precedes it are significantly destabilized at pH 7. A large number of negatively charged residues are located in this region of the protein at pH 7 (Figs. 1b and 6d). In the native state, these negative charges are shielded by the bound calcium ion. In the absence of bound calcium in the molten globule, there will be significant electrostatic repulsion between these acidic residues leading to destabilization. This effect is largest in 4SS α-LA. This is likely to arise because of the C73-C91 disulfide, which is known to be critical for calcium binding as it helps to position the ligand groups in the correct orientation.^27,28^ This long-range covalent cross-link in 4SS α-LA will restrict the conformations that can be adopted by D74, D78, D82 D83, D84, D87, and D88 in order to minimize repulsion. All-Ala and [28–111] α-LA lack this disulfide cross-link, and residues between 73 and 91 will have greater conformational freedom to adopt low energy conformations leading to a smaller destabilization at pH 7.

The differences in stability observed in this NMR study for the C-helix and the preceding residues can explain some of the discrepancies reported in the literature for the C-helix region in the α-LA molten globule. Hydrogen–deuterium amide exchange studies carried out for 4SS α-LA and [28–111] α-LA at pH 2 using NMR and mass spectrometry, respectively, show measurable protection for C-helix amides.^17,18^ In addition, the urea-induced unfolding studies at pH 2 show that the C-helix is structured in the absence of urea and that it is resistant to unfolding by urea in all three variants of α-LA.^15,21,22^ On the basis of these data, it has been concluded that the C-helix is stably formed in the molten globule at pH 2. Several previous studies carried out using the [28–111] α-LA variant at higher pH seem to contradict this observation. Insertion of a proline into the C-helix did not lead to a significant change in the overall ellipticity measured by far-UV CD.^16^ The conclusion from this proline scanning mutagenesis study was that the C-helix is not formed in the α-LA molten globule. Alanine mutagenesis studies focused on identifying important hydrophobic contacts in the molten globule found that alanine substitutions in the C-helix did not have a significant effect on the propensity to adopt a native-like fold while mutations in all other helices were found to be important.^29,30^ It was concluded that the C-helix is less structured in the α-LA molten globule. These proline and alanine variants were studied at pH 8.5. The NMR results presented here can reconcile these apparently conflicting results for the C-helix. We have shown that at pH 7, the presence of a large number of negative charges in the vicinity of residues 78–88 leads to destabilization of the C-helix region. For all-Ala α-LA, the C-helix is not fully populated in the absence of urea at pH 7 and the helix is not fully stable in [28–111] α-LA. The results for these two species, rather than the observations for 4SS α-LA, are relevant for the mutagenesis studies because mutations were made in [28–111] α-LA. At pH 2, the aspartic acid residues are not charged and this leads to an increased stability for the C-helix, which is demonstrated by the amide protection observed for 4SS and [28–111] α-LA.

The results for the L11P and Q117P variants of [28–111] α-LA at pH 7 demonstrate a difference in the importance of long-range contacts at the two pH values. Earlier studies had shown that the unfolding of the A-helix and AB loop is coupled to unfolding of the C-terminal 3_10_ helix at pH 2. In the present study, the stability of the two termini does not appear to be coupled. The electrostatic properties of the C-terminal region change significantly between pH 2 and pH 7 (Fig. 6c). At pH 2, this region has two positively charged residues, K114 and K122, but is dominated by uncharged hydrophobic and polar groups. It appears that interactions between the A-helix and residues including Q117, W118, L119, and L123 may be important for the stability of the A-helix. At pH 7, E113, E116, E121, and L123 have negatively charged carboxyl groups. The highly charged nature of the C-terminus may lead to the loss of hydrophobic contacts with the A-helix at pH 7; this would explain the uncoupling of the stabilities of the A-helix and the C-terminal 3_10_ helix observed in Q117P at pH 7. In addition, these electrostatic effects may also diminish long-range hydrophobic contacts with the B-helix. The loss of such stabilizing hydrophobic contacts at pH 7 could explain the overall lower stability of the helices of the α-domain at pH 7 compared with pH 2.

Previous studies by Kuwajima *et al.* have shown a similarity between the kinetic molten globule formed transiently during refolding at neutral pH and the low pH equilibrium molten globule, the A-state.^31–33^ These studies have focused on global parameters including helical content measured by far-UV CD, formation of tertiary structure measured by intrinsic tryptophan fluorescence, and compactness measured by small-angle X-ray scattering. As a result of these studies, the low pH molten globule has been considered to be a good model for the transient kinetic species observed during refolding. The residue-specific characterization of the pH 2 and pH 7 molten globule states of α-LA presented here has highlighted differences that exist between the two species. Most notable are differences in the stability of structure for the C-helix and the calcium-binding loop that precedes it and differences in the contribution of long-range hydrophobic contacts between the N-terminal and C-terminal regions of the α-domain to the stability of the molten globule. The electrostatic effects that influence the equilibrium molten globule at pH 7 will also affect the kinetic molten globule studied at pH 7 in refolding experiments. In this study, we demonstrate that the A-state is not identical with the equilibrium molten globule present at pH 7, and therefore, the A-state may not be the best model for the kinetic species. In contrast, the low pH A-state may provide a better model for some of the properties of the α-LA molten globule *in vivo*. In particular, the low-pH species may be relevant when considering functions involving membrane transport and calcium-triggered membrane release^34^ or the formation of HAMLET (human α-LA made lethal to tumor cells)^35^ because these species appear to be formed via exposure of α-LA to lower pH at the membrane surface or in the stomach. This study also highlights the influence that disulfide bonds have on the stability and character of the molten globule. Therefore, results from studies obtained for all-Ala or [28–111] α-LA at high pH may not be relevant to the kinetic species formed during refolding of 4SS α-LA. In addition, caution needs to be exercised when collating experimental and computational data obtained at different pH values when formulating an overall picture of the molten globule.

A close similarity between equilibrium molten globule states and kinetic species observed during refolding has been reported for several other proteins including apomyoglobin,^6,36^ cytochrome *c*,^37,38^ and ribonuclease HI.^39,40^ It is interesting to note that for these proteins, the equilibrium molten globule state is studied at a lower pH than the kinetic refolding intermediate, and therefore, the pH-dependent electrostatic effects reported here to be important for human α-LA may also be of relevance in apomyoglobin, cytochrome *c*, and ribonuclease HI.

## Materials and Methods

### Sample preparation and NMR spectroscopy

^15^N-labeled recombinant human 4SS α-LA, [28–111] α-LA, all-Ala α-LA, L11P, and Q117P were expressed and purified as described previously.^17,21,22^ NMR samples contained 0.5–1.0 mM protein at pH 7.0 in 95% H_2_O/5% ^2^H_2_O. 4SS α-LA samples contained 10 mM ethylenediaminetetraacetic acid to ensure that the protein was calcium free. 2D gradient-enhanced ^15^N–^1^H HSQC spectra (500 MHz)^41^ consisting of 128 complex *t*_1_ increments of 1024 complex data points were collected for urea concentrations ranging from 0 to 9 M at 20 °C. Sweep widths of 961.5 and 5405.4 Hz were used in the ^15^N (*F*_1_) and ^1^H (*F*_2_) dimensions, respectively. Sixteen scans were collected for each *t*_1_ increment. Resonances in the spectra were assigned using standard assignment procedures applied to ^15^N-edited 3D gradient-enhanced nuclear Overhauser enhancement spectroscopy-HSQC spectra^41,42^ collected in 6 M urea, for all-Ala α-LA, and 9 M urea, for 4SS α-LA, [28–111] α-LA, L11P, and Q117P. All NMR data were processed using Felix 2.3 (Accelrys, San Diego, CA). All HSQC spectra were contoured so that a cross peak defined by a single contour has 20–25% of the intensity of a cross peak corresponding to a fully unfolded residue. All the spectra were contoured in the same way to allow a direct comparison of the contour plots. The HSQC urea titration data are summarized in histogram format in Figs. 4 and 5. The height of each bar in the histogram represents the urea concentration at which a peak corresponding to 20–25% of the intensity of a fully unfolded peak is first observed. Data collected at pH 7 are compared with data collected previously at pH 2.^15,21,22^ The histograms in Fig. 4d–f show the difference between the urea concentrations at which a peak is first observed at pH 7 and at pH 2 ([urea]_pH 7_ − [urea]_pH 2_).

### CD spectroscopy

Far-UV CD measurements at 222 nm for the all-Ala and [28–111] variants of α-LA at pH 2 and pH 7 were carried out at 20 °C using a Jasco J720 CD spectrophotometer. The protein concentration was ∼ 15 μM, and a cuvette of 0.1 cm path length was used. The reported ellipticities are the average of 120 measurements collected over a period of 1 min. The measured ellipticities at each urea concentration were divided by the ellipticity in the absence of urea to obtain the fraction of ellipticity at 222 nm plotted in Fig. 2a. *C*_m_, the unfolding midpoint urea concentration, was determined by curve fitting as the urea concentration at which 50% of the ellipticity at 222 nm was lost. The CD data for 4SS and all-Ala α-LA at pH 2 are taken from previous studies.^15,21^

## Figures and Tables

**Table 1:** Unfolding midpoint observed in urea at pH 2 and pH 7 for 4SS, [28–111], and all-Ala α-LA

	*C*_m_ at pH 2	*C*_m_ at pH 7	Δ*C*_m_ (pH 2 − pH 7)
4SS α-LA^19^	6.9	4.6	2.3
[28–111] α-LA	5.4	4.8	0.6
all-Ala α-LA	2.9	2.3	0.6
